# Systolic ShMOLLI T1-mapping is feasible in tachyarrhythmia, with improved image quality compared to diastolic readout

**DOI:** 10.1186/1532-429X-17-S1-Q5

**Published:** 2015-02-03

**Authors:** Rohan S Wijesurendra, Alexander Liu, Barbara Casadei, Matthew D Robson, Stefan Neubauer, Vanessa M Ferreira, Stefan K Piechnik

**Affiliations:** 1 Division of Cardiovascular Medicine, University of Oxford, Oxford, UK

## Background

T1-mapping using the Shortened Modified Look-Locker Inversion Recovery (ShMOLLI) technique [1] enables assessment of myocardial characteristics, such as oedema, scar and diffuse fibrosis. However, cardiac pathology is often accompanied by tachyarrhythmia, which may cause mistriggering and inaccurate T1 estimation. We hypothesised that systolic T1-mapping may overcome this issue without significantly affecting T1 values or data quality compared to conventional diastolic T1-mapping.

## Methods

Native T1-maps were acquired using ShMOLLI at 1.5T (Siemens Avanto MR scanner). Healthy volunteers (n=10, age 36±7 years, 5 males) in normal sinus rhythm (HR 61±9 bpm) provided three short axis (basal, mid-ventricular, apical) slices to generate an AHA 16-segment model. Serial T1-maps were acquired at varying prescribed trigger delay (TD) times: 0ms, 50ms, 100ms, 150ms, 500ms (MOLLI TD [1], conventional ShMOLLI) and "end diastole" (captured TD minus 50ms). We acquired T1-maps using the conventional ShMOLLI matrix basis (Mx) of 192 and a smaller Mx=176 to reduce image readout time and sensitivity to systolic motion. T1 values and image quality using R^2^ maps were compared. Finally, the feasibility and image quality of systolic T1-mapping (defined as TD=0ms/Mx=176) was tested in 5 patients with tachyarrhythmia (n=2 sinus tachycardia, n=3 atrial fibrillation; mean HR range 110-140 bpm).

## Results

**Figure 1:**
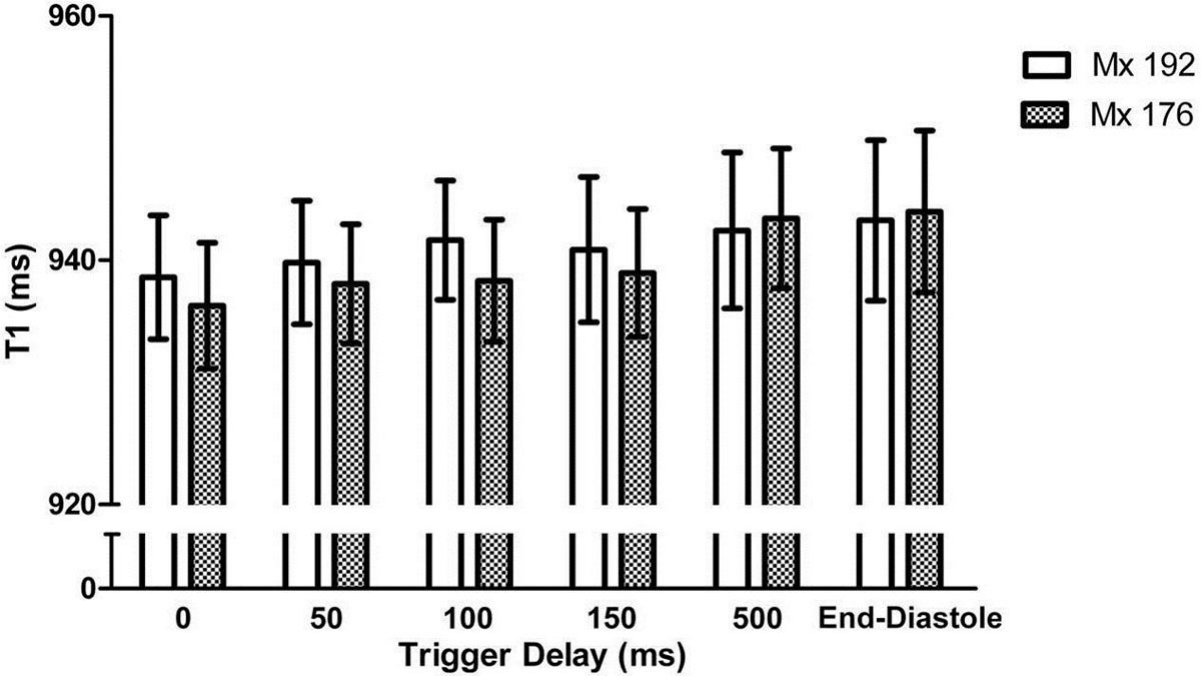
Mean segmental T1 values in normal volunteers are not significantly affected by alterations in trigger delay (TD) and matrix basis (Mx). Error bars represent 95% confidence intervals.

**Figure 2:**
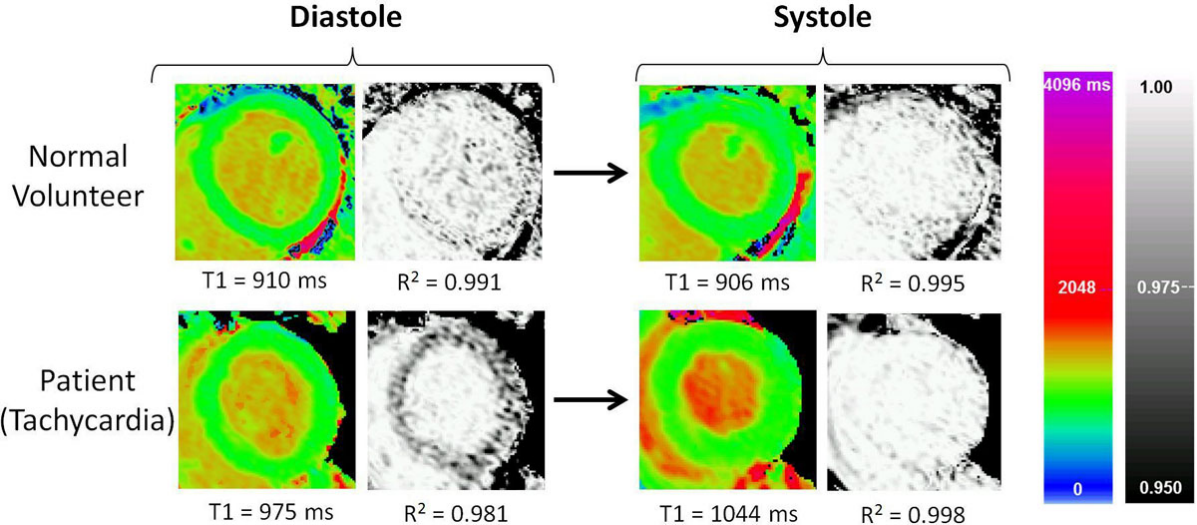
Representative T1- and R^2^-maps acquired using conventional diastolic ShMOLLI T1-mapping (TD=500ms/Mx=192) and systolic T1-mapping (TD=0ms/Mx=176). In the normal volunteer (upper panel, normal sinus rhythm), T1 and R^2^ maps have similar appearances and demonstrate near equivalence of T1 and R^2^ values. In a patient with myocardial oedema and sinus tachycardia (lower panel, mean HR 110 bpm), mistriggering of the diastolic T1 map has resulted in underestimation of T1 values and distinctively darker appearance of myocardial tissue on the R^2^ map, whereas systolic T1-mapping circumvented mistriggering with excellent T1 fit on the R^2^ map.

In normal volunteers, native T1 values were not significantly affected by TD or Mx, whether on a whole-heart, per-slice or segmental basis (Figure 1). In keeping with the results of previous MOLLI-based studies [2,3], there was a trend towards lower T1 values in systole compared with diastole. However, absolute differences were very small (maximum 8ms or 0.8% of the mean normal T1 value), comparing favourably with the ~2% overall variability of ShMOLLI T1 values [1,4]. Similarly, data quality was equally excellent using both conventional ShMOLLI diastolic readout (TD=500ms/Mx=192; median R^2^=0.996) and systolic readout (TD=0ms/Mx=176; median R^2^=0.997) (Figure 2, upper panel).

In patients with tachyarrhythmia (Figure 2, lower panel), the conventional diastolic readout (TD=500ms/Mx=192) often resulted in mistriggering and poor estimation of T1 values, whereas systolic T1-mapping (TD=0ms/Mx=176) produced consistently excellent T1-maps (median R^2^=0.999) with no mistriggering.

## Conclusions

In healthy volunteers in normal sinus rhythm, ShMOLLI T1-maps using systolic readout produce equivalent T1 values and image quality to diastolic readout. In tachyarrhythmia, systolic ShMOLLI T1-mapping circumvents mistriggering and underestimation of T1 values, providing excellent quality T1-maps. Systolic ShMOLLI T1-mapping is feasible in tachyarrhythmia and may extend clinical applicability to challenging rhythms such as atrial fibrillation, frequent ectopic heartbeats or sinus tachycardia.

## Funding

The research was supported by the National Institute for Health Research (NIHR) Oxford Biomedical Research Centre based at The Oxford University Hospitals Trust at the University of Oxford. RW acknowledges support from the BHF Centre of Research Excellence, Oxford (RE/08/004).
